# Advancing the understanding of children’s digital engagement: responsive methodologies and ethical considerations in psychological research

**DOI:** 10.3389/fpsyg.2024.1285302

**Published:** 2024-04-23

**Authors:** Natalia Kucirkova, Sonia Livingstone, Jenny Radesky

**Affiliations:** ^1^University of Stavanger, Stavanger, Norway; ^2^The Open University, Milton Keynes, United Kingdom; ^3^London School of Economics and Political Science, London, United Kingdom; ^4^University of Michigan Medical School, Ann Arbor, MI, United States

**Keywords:** technology, children, Belmont principles, ethics, psychological methods

## Abstract

Children’s perspectives and experiences with digital media (digital engagement for short) are becoming difficult to observe and measure in today’s highly multi-faceted, personalized and dynamic media ecosystem. In response, psychologists are developing a host of innovative methods. These may be broadly divided into those which prioritize children’s active participation in research and those which develop techniques for passive observation. This article presents a state-of-the-art review of emerging methodologies to highlight the ethical issues that arise, by drawing on the Belmont principles for ethical research. We identify strengths and weaknesses of both participatory and non-participatory methods and recommend ways for future research to harness the potential of child-centered, responsive, and ethical methods.

## Introduction

As artificial intelligence (AI), smart toys and devices, social media, and other technological innovations become more widespread in society and increasingly integrated into children’s lives, there is mounting interest in how psychological science can enhance understanding of their influence on or significance for children’s perspectives and experiences (Pataranutaporn et al., 2021). The affordances of modern technologies challenge psychologists to find methodologies appropriate for the diverse and complex psychological and social contexts in which these technologies are embedded and used. While there have been significant advances in collecting and processing extensive online data that reveals information about adult experiences, accompanied by specialized tutorials on data scraping (Bradley and James, 2019) and privacy guidelines for online research (Walsh et al., 2018), such methods cannot be automatically applied to children. In this paper, we reflect on how ethically to leverage data collected with or from children to understand how they might be impacted by digital media.

Psychologists need to identify responsive methods to study children’s perspectives and experiences that can effectively navigate the ethical complexities of working in a rapidly changing technological landscape with vulnerable populations. By “responsive,” we refer to methods that address the affordances of contemporary and still-evolving digital technologies. For example, portable personal devices such as smartphones and tablets allow children to engage with digital content across settings - in schools, homes, or on the move. Meanwhile, the social and interactive nature of digital interfaces (from mobile games and video-sharing platforms to EdTech products) necessitates innovative methods to capture evidence of their impact. Datafication particularly poses new research challenges, now that technologies harness users’ personal data in often-opaque ways to tailor and automate digital products and services (Hobbs, 2020), for example resulting in a personalized algorithmic content feed for each child (Kucirkova, 2019). On the other hand, while the mobility, interactivity, datafication, and personalization affordances of today’s technologies fuel continued technological innovation, they also offer new opportunities for psychological research (Flensburg and Lomborg, 2023). In this article, we are particularly concerned to recognize methods that try to capture the child’s experience/perspective of emerging digital technologies used in family contexts.

The field of psychology has undergone a notable shift in the past decade, positioning people as research participants rather than subjects, thereby acknowledging them to be reflexive agents whose experiences and perspectives merit careful attention (Woodhead and Faulkner, 2008; Mukherji and Albon, 2022). One consequence is the growth of child-centered methods, by which we mean approaches that prioritize the child’s voice, agency, and experience. While such methods may be more or less participatory or observational, as we elaborate later, they advance research goals that center on the child’s perspective, needs, and interests rather than adult-framed theoretical or even commercial goals. In relation to digital technologies, child-centered methods are valuable on two grounds. First, methods that help understand and measure what children *experience* in context, and what concerns them (in other words, their *perspectives* on these experiences) counters the temptation to assume that children’s experiences and perspectives are both homogenous and mirror those of adults. This in itself can be productive in generating findings of interest for policy, regulation, and practice; and it respects children’s right to be heard on matters that affect them. Second, in the context of complex, personalized, increasingly opaque and sometimes deceptive technological processes, adult researchers too can find it difficult to understand the consequences of digital engagement, necessitating creative methods to trace and document the range and specificity of all users’ experiences and their embedding within a wider digital ecosystem (Mallawaarachchi et al., 2023).

We conducted a state-of-the-art review (Grant and Booth, 2009) to identify and critically evaluate emerging child-centered psychology methods suitable for a fast-changing digital world. The methods we review are not device-specific: they span all levels of technology design from video games, large platforms, and immersive environments to interactive apps and educational technology, marketing data collection practices, and their interactions with the unique characteristics of children. To take just one example, consider that when an educational platform processes children’s personal data, children (and even their parents and teachers) are generally unaware of such data collection and usage, including how it may reveal and monetize children’s psychological characteristics (Day et al., 2022). For researchers to identify the consequences of data-driven educational technology for children, their methods must be responsive to its particular affordances (e.g., type of data collected, whom shared with), on the one hand, and attuned to the needs and interests of children (e.g., privacy, freedom from manipulation), on the other. At the same, time, such methods must also meet high ethical standards and recognize the power differentials inherent in a digital environment largely designed by corporations. Corporations are already collecting extensive data on children/adolescents for marketing purposes and product growth, without beneficence or respect for persons (see, e.g., recent lawsuits filed against Meta by a bipartisan coalition of attorneys general in the United States). Therefore, as researchers increasingly use novel forms of tracking or data collection, identifying community-responsive and ethical practices is crucial.

In scoping the range of emerging psychological methods, we identified a burgeoning set of both participatory and non-participatory methods. Each has its adherents and each builds on a long tradition of methodological experimentation as well as having an underpinning philosophy of social science (Bohman, 1993). Broadly speaking, participatory methods seek children’s subjective (‘insider’) expressions, typically through qualitative and consultative methods. By contrast, non-participatory or observational methods prioritize ‘outsider’ judgments by researchers and observers, often by implementing principles of experimental or quantitative design. In practice, each set of methods builds on and extends those traditionally used to research children’s perspectives (such as interviews, focus groups, consultation, and co-design) and experiences (primarily observational or experimental methods, though also parental reports of children’s experiences). As we discuss later, there is increasing interest also in hybrid methods that triangulate participatory and non-participatory methods, aiming to overcome the constraints of each to optimize the potential of emerging methods for understanding children’s digital engagement and its consequences.

By adapting research methods for digital contexts (for example, conducting interviews via videoconferencing), unique insights into children’s digital experiences and perspectives can result. But there are also pitfalls. Our aim is to present and evaluate the emerging methods for their research insights and ethical implications, recognizing that the ethical challenges that arise may require constraints on methodological innovation. To frame our analysis, we draw on the widely used Belmont principles for ethical research in psychology.

## Belmont principles

The Belmont principles encompass: *Respect for Persons* (which, in the present context, includes respect for children’s autonomy and point of view, and informed consent regarding research participation and data sharing); *Beneficence* (including the welfare of beneficiaries associated with a research study, as well as maximizing benefits for children through research designs that can inform design or policy change); and *Justice* (including equitable and inclusive community involvement to inform study design and outcomes). In what follows, we synthesize the emerging methods for researching children’s experiences and perspectives with mobile, interactive, personalized and data-driven technologies. We prioritize those non-participatory and participatory methods that aim to be responsive and child-centered, critically evaluating their ethical implications.

## Non-participatory methods

### Collecting viewing histories

To capture the sequence of videos or other digital content that children have viewed over time, researchers can request that parents or children export (or copy and paste) their recent viewing history into secure databases, after informed consent is provided (Radesky et al., 2020). The results can provide convenient and useful information that can be difficult to capture accurately through interviews, especially for viewing how content is delivered to children over time across multiple channels or devices. However, it is important to take into account the possibility of shared devices among family members who may not use personal logins to access a platform such as YouTube. Hence the data must be carefully checked to be sure who in the household watched which video and that the viewing history analyzed (including associated postings, images, or comments) does not include non-research participants. It is also important to develop mitigation plans should violent or sexual content be found in young children’s viewing histories. Also important from an ethical standpoint, researchers must be mindful that the norms and regulation of different social media platforms determine whether participants morally and legally own their data or perceive them to be private or public (Hennell et al., 2020). In assessing viewing histories, even if viewing history data are private, researchers must obtain children’s informed and ongoing consent, as highlighted by the Belmont principle of *Respect for Persons*. For example, the data collection practices used in Screenomics, in which screenshots are taken every 5 s when a smartphone is in use, may not be legal in all states or countries (Reeves et al., 2021). An alternative is to create research accounts for avatars based on real children’s interests and online behavior (Revealing Reality, 2021) and collect data on the recommendations and advertisements sent to (rather than generated directly by) those accounts.

### Passive sensing

Researchers often rely on direct observations to study children’s experiences. In digital contexts, these can now be complemented by passive sensing technologies, popular for their efficiency in data collection at scale. In this method, used by academic researchers and marketing firms, data streams from internet-connected devices, including smartphones/tablets, school devices, or smart home technologies are harnessed and analyzed. Timestamped output from passive sensors also allows researchers to examine children’s digital behaviors at any time of day or night. However, Zhang et al. (2022) found that only 10 out of 47 eligible studies considered ethical questions of informed consent when collecting passive sensing data from users’ smartphones (calls, messages, application usage). In their study of smart-home technology, Nelson and Allen (2018) highlight the researchers’ challenge of adapting not only to technological affordances but also to rapidly evolving public perceptions on permissible data practices. Zhang et al. (2022) recommend as ethical practice that the permissions granted to individual commercial applications should be strictly limited, telephone numbers should be encrypted, and Bluetooth/Wi-Fi MAC addresses and call/messages numbers anonymized, so that the data can ‘maintain uniqueness but lose traceability’ (p. 9). We concur with Zhang et al.’s (2022) recommendation: a considerable number of American citizens experience a prevailing sense of resignation regarding the utilization of their data traces by corporations and marketers. Thus, to meaningfully progress research in this area, it is crucial to align expectations regarding informed consent, data security, and anonymization.

### Data scraping from platforms and app marketplaces

Where once researchers observed children in person, emerging methods include both virtual observation and data scraping techniques, which, simply put, are techniques where a computer program retrieves information from another computer program. Davis et al. (2019) scraped the available comments and replies on two viral YouTube videos in their social identity research with adults. They highlighted the benefits of researchers engaging with publicly available datasets on social media networks, recognizing these social sites of action where multiple identities play out. Although data scraping is widely used in cyber forensic psychology as well as social media analytics (e.g., Batrinca and Treleaven, 2015), it is unlikely that child users are aware that their publicly posted social media comments can be scraped for research purposes. Belmont’s *Respect for Persons* principle reminds us that researchers should provide participants with the option to withdraw or consent to their data to be used in a different context, and shared with a different audience than that initially intended by users (Rossi et al., 2022).

Data scraping involves not only collecting usage data but also social data such as ratings, ‘Likes,’ and comments. These offer valuable insights into children’s engagement with specific apps, games, e-books, or platforms. Until now, these data have been little used in research with children. However, Meyer et al. (2019) examined the prevalence of in-app advertising to children by downloading the most frequently downloaded apps in the ‘5 and Under’ section of the Google Play Store. In a later study, Meyer et al. (2021) evaluated the quality of educational design in top-downloaded or top-rated apps from the Google and Apple app stores. In both cases, the download frequency and ranking data enabled valuable inferences about the technologies’ design and suitability for young children. However, rating data may be inflated by bot-driven downloads; it is also possible that some apps are downloaded and never used. Moreover, download data do not provide insight into how children actually use popular apps and interfaces, although such data is typically collected and monetized by the platforms (Livingstone et al., 2022). Indeed, as whistleblowers’ testimonies at US Senate hearings showed (Dyer, 2023), internal research documents from Meta detail how adolescents’ usage data are used for driving growth and product decisions, rather than bringing child-centered designs to market. As data collection methods continue to evolve, researchers will gain new opportunities to generate valuable data, necessitating careful evaluation of the potential insights and ethical concerns (Kim et al., 2022).

## Participatory methods

### Virtual interviews and ethnography

Traditionally, qualitative researchers relied heavily on face-to-face interviews, but these are now being supplemented by virtual formats, offering new research opportunities but also raising questions about the ethical principle of *Justice*. For example, using in-built audio-recording features of videoconferencing tools such as Zoom or Skype may impede participation from those with poor connectivity, and it also threatens data security (Khan and MacEachen, 2022). On the other hand, use of video chat technologies may facilitate the involvement of children who face other barriers to research participation (such as geographic distance or transportation difficulties). For example, Han (2024) used video interviews to document how Chinese left-behind children use WeChat to sustain long-distance family relationships. Some young people may prefer a Zoom interview to a face-to-face one as they feel comfortable in their own home and even like keeping the camera off (Pothong and Livingstone, 2022). However, the affordances of the digital interview may also impede the disclosure of intimate, controversial, or traumatic details, particularly if there are technical interruptions, and it can be problematic that the circumstances of the child are not visible to the interviewer.

While respect for the child’s perspective centers their perceptions and views, children often see the world through the lens of their first relationships – with their parents acting as informal guides in media literacy and their siblings as primary social partners (Dunifon et al., 2017). Thus, a range of family members may valuably participate in the research. Particularly with younger children, conceptual models and methodological approaches are needed that interrogate dyadic and group experiences of technology, recognizing that the child’s media experience is nested within multiple spheres of influence on a developing child (Bronfenbrenner, 1979; Takeuchi and Levine, 2014). Multiple research participants can join Zoom interviews, for instance, although managing the interactions online can be tricky, with norms of speaking and privacy in flux and, again, because the researchers’ knowledge of the full situation around a child is restricted.

### Digital walk-throughs

While digital walk-throughs (i.e., a participant playing a video game or looking at social platform as the research looks on) may be limited to the child “performing” what they think the researcher wants to hear, they improve upon traditional semi-structured interviews because they allow the child to react to digital stimuli, design affordances, or social interactions on the platform in real-time. Indeed, children enjoy participating in digital walk-throughs where they show researchers their digital experiences by sharing (or videorecording) their screen activities. This approach improves the ecological validity of research insights by allowing exploration of the digital spaces that children ordinarily inhabit, whether in the presence or absence of the researcher; this especially contrasts with methods which ask the child to complete contrived experimental tasks. A specific ethical challenge here is that a child’s talk-aloud commentary can include unexpected disclosures, requests for secrecy, or higher levels of confidentiality (Livingstone and Locatelli, 2014). Specifically, it can reveal unauthorized use of specific platforms or apps and there is a risk of seeing who the child is interacting with online, though those others have not consented to the research. There is even a risk that the child will show an illegal image (such as an indecent image of a child), indicating that the child might be being exploited or harmed by using a digital platform. This requires researchers to promptly respond to ensure the child’s safety. Discussion with the child’s caregivers or outside professionals might be necessary if researchers uncover the child’s exposure to harmful or inappropriate platforms, although respecting older children’s privacy and confidentiality is also important. Such risks must be planned for, and researchers should clearly delineate which data will and will not be shared with caregivers or outside professionals, usually by offering conditional rather than unconditional confidentiality, especially in contexts where mandatory reporting applies.

### Virtual role-plays

Belmont’s *Justice* principle is enhanced when psychologists treat children as competent informants [see Cater and Øverlien (2014)] able to report their experiences, for example through virtual role plays. Role-play is considered a vital play mechanism for children to express and develop their identity, as children recreate experiences, events, and aspects of their life and relive them as pretend worlds (Kingdon, 2018). This layering of pretend and real worlds is intensively experienced in digital games where the child can take on an avatar who bears resemblance to their own characteristics and use it to mirror their inner world, also revealing their understanding of the game (Stenros and Sihvonen, 2020). If researchers create avatars to be part of children’s role-play and digital games, they become part of the identity construction in children’s fictional story-worlds. A certain level of deception may be necessary for researchers to be able to authentically assess the systems children participate in. Seeing a researcher directly engage in a physical role-play is, arguably, less deceptive than researchers adopting virtual avatars. Researchers therefore need to take extra care to get the child’s ongoing and fully informed consent if adopting avatars in digital games.

### Virtual co-design

A method aligned with Belmont’s *Beneficence* principle is adult-child technology co-design, increasingly performed virtually. In-person co-design with children is often constrained in terms of number and diversity of children involved, limiting researchers’ ability to ensure diversity-informed and culturally attuned insights into digital design. Virtual co-design and virtual co-design panels address this challenge as they provide access to the same materials and conditions for participation for all users, potentially at scale. However, virtual co-designs can be challenging as researchers may be less familiar with the local conditions or cultural norms. For example, a social robot co-design study with teenagers showed how trust and transparency vary in digital and in-person co-design, with less trust and transparency online (Björling and Rose, 2019).

A related example concerns virtual consultation panels with children. Here, data collection typically involves both digital and in person methods (e.g., face-to-face discussion is preceded by an online panel discussion). Whether researchers solicit views from specialist child panels or recruit children through teachers, parents or professionals who work with children, they increase the opportunities of consulting diverse children in a shorter timeframe but also the ethical challenge of building consensus among disparate perspectives (Dinnebeil et al., 2006).

## Hybrid methods

The hybrid methods we present next suggest valuable strategies for addressing the challenges noted with single methods.

### Multi-method digital toolkits

The Comprehensive Assessment of Family Media Exposure Consortium developed a tool that combines a web-based questionnaire and time-use diary directly completed by the child with a passive-sensing app installed on family mobile devices, in an effort comprehensively to measure a child’s screen time exposure (Barr et al., 2020). The tool records the type of technology used (e.g., TV, PCs, tablets, and smartphones), the available software (digital games, apps, and e-books), purposes of use (e.g., education and emotion regulation), and situational or relational context of use (e.g., bedtime, with or without caregivers present). Passive sensing by Android mobile devices assesses which apps are used, for how long, and at what time of day. This information can be overlaid onto time diary data to examine the context of screen use. In this way, researchers can explore whether, for example, siblings or caregivers co-use educational technologies with young children, or whether portable technologies are used outside (e.g., geocaching, nature identification apps) or the community (e.g., Pokemon Go). This method requires collection and integration of multiple digital data streams, which must be processed and stored securely in accordance with ethical and data protection regulations. As such, the method addresses the mobile, interactive, and personalized character of modern technologies efficiently and effectively and can be considered responsive. Through such a hybrid approach, the limitations of passive sensing apps (for example, not knowing what content was experienced via social media or video-sharing or, even, whether the device was left on while the user was sleeping or absent) can be addressed by triangulating with participatory methods.

### Children’s creative digital experiences followed by quantitative coding

Some methods enable children to participate actively by using technologies to document their lives (e.g., Hov and Neegard, 2020 with GoPro cameras), to read and create digital stories (e.g., Kucirkova and Flewitt, 2022), or for role play such as caring for digital pets (e.g., Marsh, 2019). Such methods capture the dynamic nature of children’s cross-media interactions and the active role children play in how the technologies respond to their creative interactions. As such, the methods have high ecological validity by collecting a considerable degree of detail in real-life contexts while also reflecting the child’s lived experiences including their decisions about where to point the camera or how to curate the data generated. However, the methods can be time-consuming to conduct, and they result inconsiderable volumes of relatively unstructured data that can be challenging to analyze. Ethical problems can arise too, including safeguarding the participants’ privacy. One possible solution is to video-record children’s interactions with a researcher-designed platform that has restricted degrees of freedom to perform actions and thus introduces predictable phases of interaction which can be coded with standardized engagement metrics. Neumann et al. (2023) followed this approach in studying children’s engagement with a specially designed social robot in pre-schools, coding for behavioral and emotional engagement with a human versus social robot instructor.

## Ethical research through inclusive practices

In conclusion, recognizing the highly individualized experiences afforded by digital media, we have demonstrated how psychological science can advance methodologically and ethically to understand children’s experiences and perceptions of rapidly evolving, mobile, interactive, data-driven personalized digital environments in everyday life contexts. This advancement is crucial for psychologists to offer valuable and valid insights into children’s technology use amidst the rapidly innovating, globalizing and commercializing nature of their digital worlds. However, the transition to virtual methods carries with it new ethical challenges concerned with privacy and data protection, revelations of child (online) harm, and deception, among other issues. Our evaluation suggests that participatory methods are more often responsive and child-centered than non-participatory ones. However, hybrid methods appear to strike the best balance between responsiveness, child-centeredness, and adherence to ethical research principles such as the Belmont principles.

As ever, it is also important that psychologists follow representative, inclusive, and not coercive recruitment procedures, with particular attention to user groups historically underrepresented in research and technology design. The strengths and limitations of survey methods change as technology evolves. Limitations include difficulty measuring aspects of human-computer interaction that participants may not be consciously aware of, for example data collection or persuasive nudges. With notable exceptions of government-run surveys or qualitative studies that focus on underrepresented groups (e.g., Mendelsohn et al., 2008), the typical approach of recruiting families through university registries, local organizations, or social networks has relied on parenting practices and perspectives that emphasized the middle-class white experience and neglected racial-ethnic groups. In addition, there is an assumption of neurotypicality in studies of psychological effects (e.g., studies of how media cause “ADHD symptoms”) without considering children’s underlying differences or including children with prior diagnoses or special educational needs (Beyens et al., 2018). Examination of the differing risk/opportunity balance in diverse subpopulations is important in ensuring that methods that work for one community do not accidentally reduce the opportunities of other communities. The process will be facilitated by increasing representation of diverse backgrounds and characteristics among psychology researchers (Vasquez et al., 2006), and expanding the range of topics and perspectives they study and represent (Cundiff, 2012).

Ethical concerns can become especially urgent in digital contexts characterized by personal data, particularly when these contexts depend on data used by researchers to generate new insights about an entire population. In particular, if research-based data sources are used by researchers to train machine learning algorithms or generative AI systems, the researchers must ensure the data represent diverse children and families. Data collection methods should be adapted according to the extent of algorithmic fairness embedded in AI-based technologies to ensure cultural sensitivity and validity. Underrepresented and marginalized communities have experienced a disproportionate level of negative experiences with modern technology, including inequitable high-speed internet access (Ramsetty and Adams, 2020), inappropriate racial-ethnic representations on video-sharing platforms (Rollins et al., 2022), and algorithms trained on biased data (Buolamwini and Gebru, 2018). Researchers should therefore consult with diverse community members with the goal of checking, for instance, the extent of algorithmic fairness embedded in AI tutoring and learning systems developed for children. Without designing measures and analyses that reflect the digital experiences of historically marginalized communities, psychological science risks generating results that center the experiences of privileged families. Already, the science of learning, underscores the importance of employing diverse methods and carefully selecting the most appropriate method for a specific context, research question, and increasingly, digital environment (Kucirkova et al., 2023). Being mindful of this diversity and choosing methodologies that best align with the research problem not only demonstrates methodological rigor but also fulfills an ethical obligation to address pressing questions regarding digital media. Given the heightened public interest in screen use for children, it is imperative to employ methodologies that yield comprehensive answers to these inquiries.

Finally, it is important that the commercial interests that drive the design and utilization of popular children’s technologies do not also shape the research agenda in the interests of business. Academic researchers find themselves increasingly competing with industry researchers and market researchers when disseminating their findings or seeking to inform policy deliberation regarding intervention, education or regulation relating to children’s engagement with innovative technologies. In addition to vigilance over research independence, funding sources and conflicts of interest, these changing circumstances invite collaborations among psychologists and researchers from other disciplines as well as with children and young people, and their caregivers and communities, to center children’s best interests and the wider public interest in advancing understanding of children’s digital lives.

## Author contributions

NK: Writing – original draft, Writing – review & editing. SL: Writing – original draft, Writing – review & editing. JR: Writing – original draft, Writing – review & editing.
